# Gliricidia (Gliricidia sepium) Green Manures as a Potential Source of N for Maize Production in the Tropics

**DOI:** 10.1100/tsw.2001.300

**Published:** 2001-11-22

**Authors:** Abdul R. Bah, Zaharah A. Rahman

**Affiliations:** Department of Land Management, Universiti Putra Malaysia, Selangor DE, Malaysia

**Keywords:** alley cropping, decomposition, fresh green leaves, incorporation, leaching, mulching, N derived from treatments, nitrogen release, subsoil, topsoil, Ultisol

## Abstract

Use of cheap, N-rich, and environmentally benign legume green manures to correct N deficiency in infertile soils is a very attractive option in the humid tropics. Understanding the influence of management and climate on their effectiveness, and quantifying their contribution to crop productivity, is therefore crucial for technology adoption and adaptation. Mineral N buildup and the contribution to N uptake in maize were studied in an Ultisol amended with fresh Gliricidia leaves. Net mineral N accumulation was compared in mulched and incorporated treatments in a field incubation study. The 15N isotope dilution technique was used to quantify N supplied to maize by Gliricidia leaves in an alley cropping. Mineral N accumulation was slow, but was much greater after incorporation than after mulching. Also, N buildup was always higher in the topsoil (0 to 10 cm) than in the subsoil (10 to 20 cm). More NO_3_-N was leached than NH_4_-N, and the effect was greater in the incorporated treatment. Surface-applied Gliricidia leaves significantly increased N uptake by maize, and supplied >30% of the total N in the stover and >20% of that in the corn grain, even in the presence of hedgerows. Thus Gliricidia leaf mulch has immense potential to improve productivity in tropical soils.

## INTRODUCTION

Soils in the tropics are predominantly highly weathered Ultisols and Oxisols, which are known to be acid in reaction and inherently infertile. Therefore, problems of heavy metal toxicities (Al) and nutrient deficiencies, especially of N and P, are very widespread[1]. Hence, management of nutrients in these soils entails significant input of mineral fertilizers. Apart from the prohibitive costs involved, this option is fraught with distribution problems and the utilization efficiencies are often very low because of high losses, especially of N, associated with the heavy rainfall conditions prevalent in these areas. On the other hand, cheaper traditional alternatives like shifting cultivation, wherein land is cultivated for a few years then left to fallow for longer periods whilst another piece of land is cultivated, are now untenable. This is because increased demographic pressure has resulted in either extremely short fallow periods or sometimes none at all[2].

However, the practice of incorporating organic residues, mainly legume green leaf manures (GLMs), in tropical nutrient management systems has been shown to be quite promising[3,4,5]. In particular, the alley cropping system in which food crops are intercropped with leguminous trees/shrubs in alleys has been found to be suitable for the uplands[6,7]. The legume hedgerows are pruned at appropriate time intervals to minimize competition effects, and the prunings are applied to provide nutrients for the crop. This system has been reported to improve soil fertility, enhance nutrient utilization, and also be very effective in soil conservation[7,8,9]. Legume GLMs can also alleviate heavy metal toxicities[10].

It has been established that the kinetics of nutrient release from decomposing GLMs are largely dependent on their biochemical characteristics, especially N, lignin, and polyphenol concentrations[11,12], as well as environmental factors like precipitation, temperature, and soil conditions[10]. As N is an important nutrient that is limiting crop performance in tropical soils, it is desirable to make this technology available to resource-poor farmers. To facilitate this, there is a need to quantify GLM contribution to N uptake and utilization, and also determine suitable methods for maximum benefit. In this regard, two experiments — a field incubation study and an alley cropping trial — were carried out with the following objectives: (1) to determine the effect of method of application on the release, accumulation, and loss of N from Gliricidia green leaves, and (2) to quantify the contribution of these leaves to N uptake by corn in an alley cropping using $^{15}N$ isotope dilution technique.

## EXPERIMENTAL METHODS

The trials were carried out at the Puchong experimental site of the Department of Land Management, Universiti Putra Malaysia. The climate here is humid and tropical with a mean minimum temperature of 19.0°C and a maximum of 32.5°C. Overall, mean annual rainfall is around 2100 mm and the relative humidity is almost always above 95%. Total rainfall during the incubation study was about 530 mm in about 40 rain events, and the pattern was one of increasing intensity with time. Soils in this area belong to the Bungor series (Typic Paleudult, clayey, kaolinitic, and isohyperthermic) and the landform is generally undulating with grasses, mostly *Pennisetum* and *Imperata* species, forming the bulk of the natural vegetation. Some properties of the topsoil (0 to 20 cm) are:

- $pH_{water}$ (1:2.5) 4.6
- $pH_{KCl}$ (1:2.5) 3.9
- 14.8 g $kg^{-1}$ organic C
- 600 mg $kg^{-1}$ total N
- 4.6 mg $kg^{-1}$ Bray-1 P

Cation exchange capacity is 8.3 cmol (+) $kg^{-1}$ with exchangeable K, Ca, and Mg values of 0.17, 0.32, and 0.18 cmol(+) $kg^{-1}$, respectively. The organic residues used were fresh green leaves of *Gliricidia sepium* (760 g moisture $kg^{-1}$) with the following biochemical constituents in per kilogram dry matter: 42 g N, 115 g lignin, 18 g polyphenol, 401 g C, 9 g Ca, 26 g K, and 2 g P.

### Incubation Study

In this case, PVC tubes (diameter 8 cm, length 25 cm) were first driven to a depth of 20 cm in the soil and then pulled out carefully with the enclosed soil. Resin bags (~150-mm mesh) containing 25 g of amberlite IR-120 cation and amberlite IRA-402 anion resins (16 to 50 mesh) were placed at the bottom of the tubes, and then replaced back into the soil leaving 5 cm of each tube above ground level. After this, 50 g of the Gliricidia GLMs were either applied as mulch or incorporated into the top 10 cm of the enclosed soil. This is equivalent to 99.5 t GLM $ha^{-1}$ and about $1 \times 10^3$ kg N $ha^{-1}$. The area was kept free of weeds by regular manual weeding. Soil and resin samples were randomly collected in four replications at intervals of 3, 5, 10, 20, 30, 40, 50, 60, and 70 days after establishment (DAE) for mineral N analysis. This involved excavating the tubes and separating the soil into two layers: 0 to 10 and 10 to 20 cm, representing the top- and subsoil, respectively. At the same time, each resin bag was removed, cleaned with a soft brush, and then rinsed in distilled water (1 min). This was then air-dried prior to chemical analysis.

### Alley Cropping Trial

Alleys were formed with *G. sepium* hedgerows 3 m apart and 0.5 m within rows. The length of each plot was 5 m and the distance separating adjacent plots was 1 m. Treatments consist of Gliricidia leaf prunings applied to plots with or without hedgerows, and to similar plots with no pruning application as the control. All were arranged in a randomized complete block design in four replications with adjacent blocks set at 2 m apart. Sweet corn was planted at intra- and inter-row spacings of 0.25 and 0.75 m, respectively. The trees in the hedgerows were pruned at a height of 1 m and the prunings applied at a rate equivalent to 160 kg N $ha^{-1}$ in two splits at 3 and 6 weeks after planting (WAP). Equivalent rates of N, P, and K were added as ammonium sulphate, triple super phosphate (TSP), and muriate of potash (MOP) to plots without pruning application. Similarly, supplementary P and K were also added in the pruning treatments. Also, $^{15}N$ labeled ammonium sulphate (10% atom excess) was added to microplots (1.5 $m^2$) in the central row of each plot (seven plants) at a rate of 40 kg N $ha^{-1}$. The plots were appropriately managed (weeding, irrigation, etc.) manually. The corn was grown for 2.5 months and harvested at physiological maturity by cutting the plants at the base close to ground level. Three central plants from the microplots were harvested, and the fresh and dry weights (70°C) of the corn grains and stover were separately recorded. Total plot yield was obtained by harvesting the remaining rows minus the border rows (0.5 m on either side).

### Chemical Analysis

Fresh soil samples from the incubation study were analyzed for $NH_4$-N and $NO_3$-N[13], and mineral N was approximated as the sum of the two. The resins were eluted with 1 M NaCl (1:4 ratio) and mineral N similarly determined. Prior to this, soil from the site was characterized using standard laboratory methods[14]. Biochemical characterization of the Gliricidia leaves was done using methods described by Anderson and Ingram[15]. Total N in the corn plant samples was determined by the Kjeldahl method following digestion with a mixture of salicylic acid-$H_2SO_4$. $^{15}N$ in the samples was determined by mass spectrometery.

### Data Analysis

Net change in mineral N was the difference between consecutive samplings. N derived from the treatments (NdfT) was calculated on the basis of the isotope dilution principle:

$$\%\ \text{NdfT} = 1 - (^{15}\text{N atomic excess in plant part in treatment plot} \div {}^{15}\text{N atomic excess in plant part in control}) \times 100 \quad (1)$$

where Control = Plots without prunings and/or hedgerows, but received N fertilizer.

The influence of time on mineral N accumulated/leached or of treatments on corn yield and N uptake was determined by ANOVA using the statistical analysis system software[16]. Comparison of means was done by the least significant difference method (LSD) for mineral N, and by Duncan's Multiple Range Test (DMRT) for the corn crop, both at the 5% level of significance.

## RESULTS AND DISCUSSION

### Accumulation and Loss of Mineral N during Incubation

Mineral N accumulation was the net result of N release from the decomposing GLM plus that from the particulate organic matter fraction in the soil (POM)[11,12,17], and loss through leaching, $NH_3$ volatilization, and/or denitrification of $NO_3$-N. Build-up occurred in two phases within 20 days of mulching the soil with Gliricidia leaves. In the topsoil, a quick build-up, due to rapid decomposition of the nutrient-rich fresh leaves, reached a first maximum value at 3 DAE - >100 mg N $kg^{-1}$ (~160 kg N $ha^{-1}$), and then a much smaller value of ~60 mg N $kg^{-1}$ (~90 kg N $ha^{-1}$) at 20 DAE (Fig. 1). The buildup in the two soil layers were equivalent in the first 10 days, and largely declined thereafter as leaching losses increased with rainfall intensity. Incorporating the leaves caused slower mineral N accretion in the topsoil, but this resulted in a much higher maximum of ~250 mg N $kg^{-1}$ (~390 kg N $ha^{-1}$) after 10 days. This also declined to a more or less constant minimum value from 30 DAE onwards. The pattern was similar in the subsoil, albeit with much lower values. The fast buildup in mineral N is consistent with the rapid leaching of soluble nutrients that reportedly occur in the initial stages of residue decomposition[4,18,19]. Later on, this slowed down conspicuously when the soluble nutrients became depleted and the recalcitrance of the substrate increased.

Congruent with the results from other studies, mineral N accretion was greater after GLM incorporation compared to mulching (Fig. 1), mostly because of greater loss of N in the gaseous form coupled with slower release rates in the latter[20]. Also, more intimate contact between the residues and the physical and biological promoters of decomposition is afforded by incorporation. Moreover, leaching could have been enhanced under the mulch because of greater infiltration emanating from

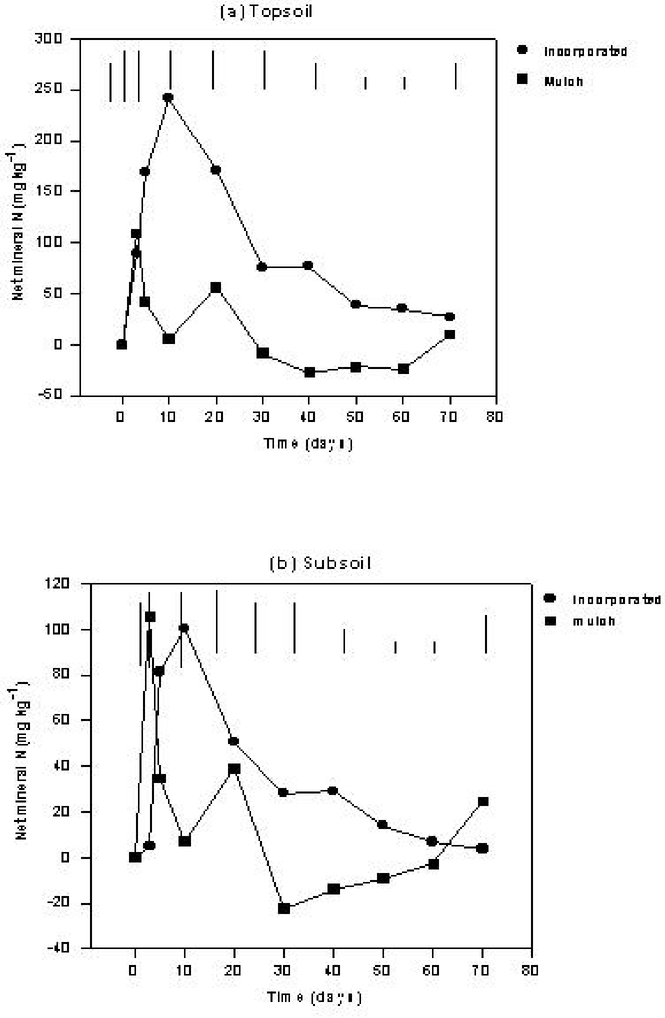

**FIGURE 1.** Soil mineral N changes in an Ultisol amended with green leaves of *G. sepium*. Net mineral N = difference between successive samplings. (Bars = $LSD_{0.05}$)

increased burrowing activities of soil macrofauna[21]. Thus, there was, on average, greater mineral N accrual in the topsoil during the first few days of mulching, but more pronounced depletion at later stages due to these losses, compared to the subsoil.

In general, a greater proportion of the mineral N leached from the top 20 cm of soil was in the nitrate form because of its greater mobility. And nitrate loss after GLM incorporation was about twice that of the surface-applied treatment (Fig. 2). This suggests that N loss under mulching could have been mainly through gaseous release. Nonetheless, there was an early flush of $NO_3$-N under mulching at 3 DAE (>200 mg N $kg^{-1}$) following the first rains, but this declined to about 50 mg N $kg^{-1}$ (~100 kg N $ha^{-1}$) at 10 DAE. Another increase to almost 200 mg N $kg^{-1}$ (~400 kg N $ha^{-1}$) occurred between 30 and 40 DAE, and declined thereafter to a minimum level (<50 mg N $kg^{-1}$). By contrast, the leaching of the mineral N components from the top 20 cm of the soil following incorporation of Gliricidia increased with time (Fig. 2). The amount of nitrate leached out increased to a maximum of about 400 mg N $kg^{-1}$ (~800 kg N $ha^{-1}$) at 50 DAE and beyond. Surprisingly, leaching of ammonium-N from this soil depth (20 cm) also exhibited an increasing trend to nearly 200 mg N $kg^{-1}$. This was probably related to the CEC[20] of the soil being too low to accommodate the high amounts of $NH_4^+$ released by the decomposing material. Despite this, the total amount of $NO_3$-N leached was still greater than that of $NH_4$-N.

## Growth of Corn in an Alley Cropping with Gliricidia

Corn grown in plots with no pruning application gave significantly higher dry matter yields than the ones amended with

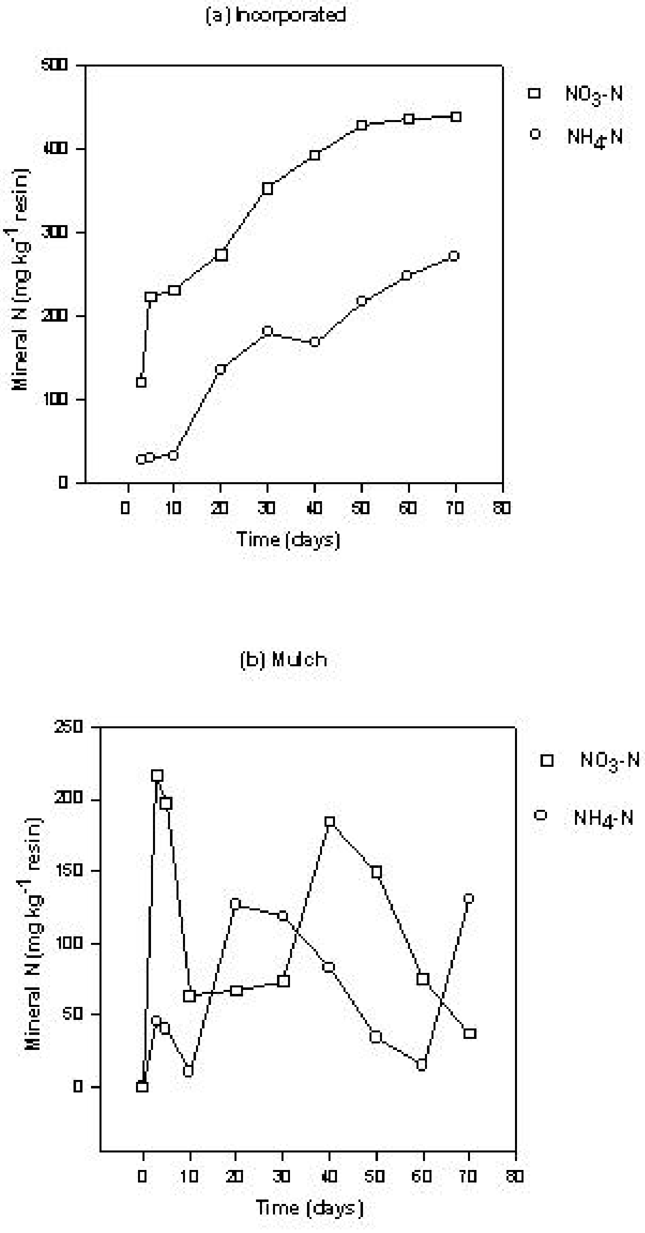

**FIGURE 2.** Leached N collected in resins following amendment of an Ultisol with GLM of *G. sepium* (calculated as amount of N in resins at each sampling).

prunings, even in the presence of hedgerows (Fig. 3). This is expected because the plots without prunings were amended with soluble fertilizers. The readily available nutrients could have even masked competition effects from the hedgerows. Hence, the most significant effect was obtained in the control within hedgerows. Nonetheless, the benefit of applying the high quality Gliricidia prunings was clearly demonstrated in the trends of total N taken up by the corn crop. The GLM showed comparable importance as a source of N with the soluble fertilizer. Thus, the leaf prunings, even in the presence of hedgerows, induced markedly higher N uptake values than the plots that received soluble N fertilizer instead of prunings (Fig. 3). These leaves have a C-to-N ratio of <10 and a polyphenol-to-N ratio of 0.4, which is less than the critical value (0.5) at which N release from residues is inhibited[12]. Therefore, the rapid release of N from the decomposing Gliricidia GLM accounts for the positive effect of the prunings. This effect was further enhanced by the fact that the prunings were applied in two splits at 3 and 6 WAP, and so could have been in synchrony with crop demand[19,22]. This was consistent with the time of greatest demand for N in maize (between 30 and 60 days after planting)[23]. Therefore, the leaf prunings alone or even within hedgerows made the highest significant contribution to N uptake. This was about 32 and 36% of N in the stover and corn grain, respectively, in plots without hedgerows based on a control that received only N fertilizer. The corresponding values for plants within hedgerows were lower, but not significantly different (25% in stover and 23% in corn grain). In general, N accumulated in the corn grains was lower than the stover for corresponding treatments, but the trends in treatment effects were coincident.

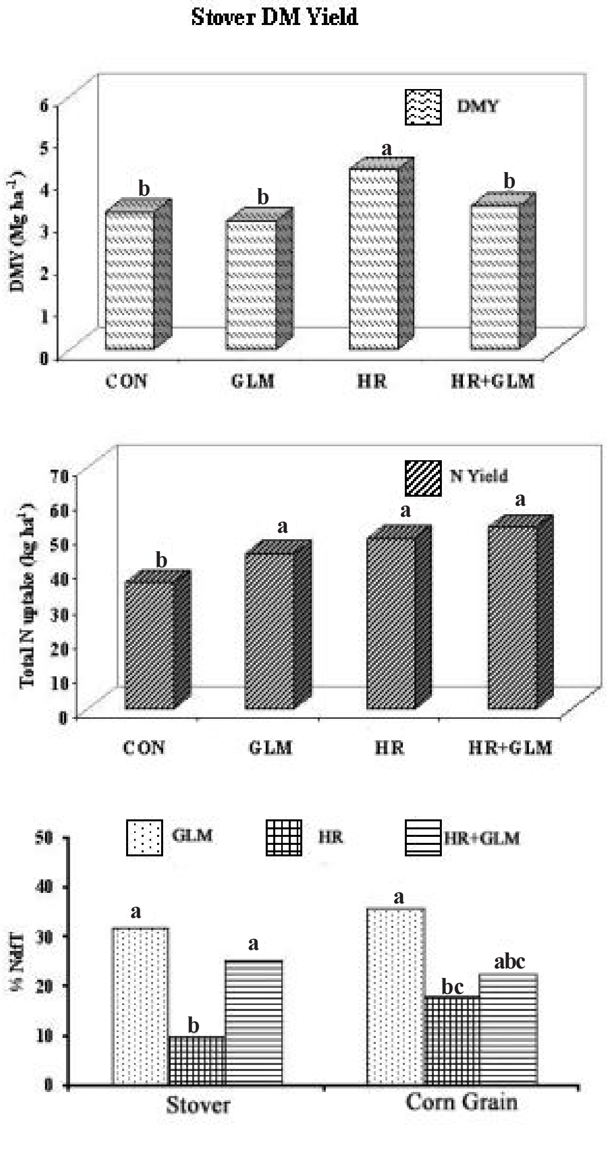

**FIGURE 3.** Dry matter yield and N uptake of sweet corn in an alley cropping with *G. sepium*.

## CONCLUSIONS

These studies demonstrate the tremendous potential of the high-quality Gliricidia GLM in the management of N in acid infertile soils of the tropics. The leaves improved N uptake in sweet corn and showed that competition from the hedgerows cannot be important in an alley cropping as long as the nutrients are sufficiently available at the time of crop demand. Because of the rapid decomposition of the Gliricidia GLM, split application at 3 and 6 WAP proved to be a suitable management strategy for corn. Although N accumulation is greater when the residues were incorporated, the possibility of high loss of $NH_4$-N in soils with small CEC and the high labour costs involved during application might limit its adoptability. Consequently, there is a need to compare the economics of GLM application in cropping systems to make an appropriate choice between mulching and incorporation.

## ACKNOWLEDGEMENTS

We very much appreciate the technical assistance of Ms. Zabedah Tumirin and Mr. Junaidi Jafaar during the course of the studies.

| | | |
|---|---|---|
| Received: | July | 18, 2001 |
| Revised: | October | 10, 2001 |
| Accepted: | October | 15, 2001 |
| Published: | November | 22, 2001 |
